# Influence of clusterin genetic variants on IOP elevation in pseudoexfoliation syndrome and pseudoexfoliative glaucoma in Turkish population

**DOI:** 10.1186/s12886-023-02850-3

**Published:** 2023-03-23

**Authors:** Birsen Can Demirdöğen, Sinem Demirkaya-Budak

**Affiliations:** Department of Biomedical Engineering, TOBB University of Economics and Technology, Söğütözü 06560 Ankara, Turkey

**Keywords:** Biomarker, Chaperone, Polymorphism, SNP, Susceptibility, Variation

## Abstract

**Purpose:**

Pseudoexfoliation syndrome (PEX) is distinguished by the deposition of fibrillary material within the aqueous humor and, in most cases, causes pseudoexfoliative glaucoma (PEG). The pathophysiologies of PEX and PEG are not completely explained. Therefore, this study aimed to assess the potential relationship between single nucleotide polymorphisms (SNPs) in the 3ʹ untranslated region or introns of the clusterin gene (*CLU*) and the susceptibility to developing PEG or PEX.

**Methods:**

Two hundred and forty patients with PEX, 239 patients with PEG, and 240 control subjects were included. Genotyping was carried out using real-time PCR (rs2279590 C/T and rs1532278 C/T) or PCR followed by restriction endonuclease digestion (rs11136000 C/T and rs3087554 T/C).

**Results:**

The minor alleles or genotypes of *CLU* SNPs were not significantly associated with PEX or PEG. IOP values of patients with PEX carrying the homozygote polymorphic TT genotype were significantly elevated compared with PEX cases with the CT or CC genotypes for rs2279590, rs11136000 and rs1532278 (*P* = .009, *P* = .007, *P* = .010, respectively).

**Conclusion:**

We present the first evidence that three SNPs in *CLU* gene (rs2279590, rs11136000 and rs1532278) might induce a rise in IOP in patients with PEX, conferring susceptibility to develop PEG.

## Introduction

Pseudoexfoliation syndrome (PEX; OMIM: 177,650) is defined as a generalized age-related disorder manifesting itself mainly in the eye. Deposition of small, white fibrillar material is observed in various intraocular as well as extraocular tissues [1]. The chronic deposition of this abnormal material in the anterior chamber of the eye leads to elevated outflow resistance resulting from the blockage of the canals that cause drainage of the aqueous humor, thus, causing a rise in the intraocular pressure (IOP). Hence, PEX is the most prevalent detectable cause of pseudoexfoliative glaucoma (PEG), a severe and chronic type of secondary open-angle glaucoma [2]. PEG prognosis is worse than that of primary open-angle glaucoma; the IOP values in PEG are often much higher and fluctuate during the day, optic nerve damage is more severe, and it is more resistant to pharmaceutical therapy [3].

The worldwide prevalence of PEX is approximately 10–20% among individuals > 60 years old [4]. The probability of a PEX patient to develop PEG within 15 years after being diagnosed with PEX has been found to be 44% [5]. However, some patients with PEX never develop glaucoma. Despite much research, the definite pathophysiological mechanisms controlling the development of PEX and advancement to PEG are yet elusive. Molecular factors that cause susceptibility to PEX and PEG should be identified for the early detection and effective management of these conditions.

Clusterin (CLU; apolipoprotein J) is a ubiquitous multifunctional protein expressed in nearly all tissues, including the iris, aqueous and vitreous humors, retina, lens, and cornea [6]. CLU is involved in various biological processes and its expression is upregulated under stressful conditions, including apoptosis, neurodegeneration, injury, and oxidative stress [7]. Moreover, it plays a molecular chaperone role to refold any damaged or misfolded proteins [8]. Notably, CLU is typically downregulated in the iris of individuals suffering PEX [3, 9, 10]. Moreover, the CLU level in the aqueous humor of these patients is surprisingly low [10]. In contrast, the anterior chamber becomes oxidative during PEX, and this oxidative environment is thought to cause the development of the extracellular granular deposits [11]. Moreover, it is known that *CLU* gene expression is induced under oxidative stress [12]. Hence, the low CLU level despite the oxidative stress suggests that some other factors are involved in the pathology. These factors may include single nucleotide polymorphisms (SNPs) within the *CLU* gene, causing decreased expression of the gene or affecting the protein structure [13–15].

The human *CLU* gene is mapped to chromosome 8p21–p12, spans over 16 kb and has nine exons. The frequencies of the minor *CLU* alleles of missense variations are very low, the global frequencies ranging between 0.0002 and 0.0653 [16]. On the other hand, the frequency of variants in introns or 3ʹ untranslated region (UTR) can be much higher [16]. Intronic polymorphisms can disrupt the recognition sequences used during pre-mRNA splicing, lowering the splicing fidelity and efficiency, and thus hampering the protein production. SNPs found in the UTR regions of a gene can also affect the gene expression by generating alternative polyadenylation signals or hindering the microRNA-mediated regulation of the gene [17]. Some of these SNPs have previously been studied in the context of PEX and/or PEG, but the results have been rather complex [15, 18–23].

It is known that distribution of genetic variations can significantly differ between populations, and that different genetic and epigenetic backgrounds, as well as lifestyle habits can modify protein expression levels. Therefore, in this study, we aimed to determine the association of four SNPs (rs2279590 C/T, rs11136000 C/T, rs1532278 C/T, and rs3087554 T/C) in the *CLU* gene with the risk of developing PEG or PEX in the Turkish population. In addition, possible association of the *CLU* SNPs with certain visual parameters including IOP was analyzed.

## Methods

### Subjects

This study included 240 patients with PEX, 239 patients with PEG, and 240 controls. Subjects’ recruitment was performed consecutively with the collaboration of Ophthalmology Department of Gülhane Training and Research Hospital, Ankara, Turkey. All the subjects of this study were adults from admixed population of Turkey and were unrelated to each other. Diagnosis of PEX or PEG was performed by an ophthalmologist from Ophthalmology Department of Gülhane Training and Research Hospital, Ankara, Turkey, as follows: Patients with PEX were identified by the presence of the characteristic fibrillary material at the lens capsule or pupillary margin during the biomicroscopic examination. Subjects were classified as having PEG if they showed typical glaucomatous optic nerve damage and visual field loss, and when the PEX findings in the anterior segment coexisted with an IOP over 21 mmHg without any pharmaceutical therapy or less than 21 mmHg with therapy. All the patients with PEG recruited in this study were on IOP-lowering medication. The control group was composed of age-matched subjects who had no characteristic fibrillar material at the pupil margin or on the anterior lens capsule during dilated anterior chamber examination, a normal optic nerve head presentation, and no defects in visual field. Visual acuity testing for each subject was performed using Snellen eye charts. Goldmann applanation tonometry, dilated direct fundoscopy and slit-lamp biomicroscopy examinations, and visual field examination using the 30–2 Swedish Interactive Threshold Algorithm with the Humphrey field analyzer (San Leandro, CA, USA) were also performed. The data for one eye was randomly included into the study in the bilateral cases. Subjects with other ophthalmologic disorders, such as hereditary retinopathy, keratoconus, uveitis, age-related macular degeneration, or pseudophakia, kidney disease, any other systemic fibrotic disorder, or any disease that could alter the visual field results. The subjects who had been under treatment with diuretics, non-steroidal anti-inflammatory drugs, or vitamins E or C were not included because they can influence IOP values.

### Genotyping for CLU SNPs

Peripheral whole blood was used to extract genomic DNA using a salting out method. GRCh38.p12 (Genome Reference Consortium Human Build 38 patch release 12) was used for variant annotations. Genotyping for *CLU* rs11136000 C/T and rs3087554 T/C SNPs were performed using a conventional PCR followed by restriction by endonuclease enzymes. The primers used for rs11136000 C/T were forward, 5ʹ CAT CTT CCA AAG CAG GCT G 3ʹ and reverse, 5ʹ CCT GAC CCC AAG TAA TAT GC 3ʹ. The primer used for rs3087554 T/C were forward, 5ʹ AGA TTG TCG CAC CTT GGT CA 3ʹ and reverse, 5ʹ TGT GAG CTG ATC GCT TGG AG 3ʹ. The PCR reagent mixture contained 20 pmol of each primer, 200 μM dNTPs, 2 mM MgCl_2_ for rs11136000 C/T, 1.5 mM MgCl_2_ for rs3087554 T/C, 1.25 U of *Taq polymerase* and 200 ng genomic DNA, in a 50 μl total volume.

The thermal cycler program was: initial 3 min denaturation at 94 °C, 40 cycles of 30 s denaturation at 95 °C, 20 s annealing at 60 °C for rs11136000 C/T or at 59 °C for rs3087554 T/C, and 50 s elongation at 72 °C, and ultimate extension at 72 °C for 10 min. In order to genotype for the rs11136000 C/T polymorphism, 246-bp long PCR products were incubated with 5 U *ApoI* at 50 °C for 40 min. This digestion reaction gave 131-bp and 115-bp long pieces for the T allele, and a noncut 246-bp fragment for the C allele. To determine the genotypes for the rs3087554 T/C polymorphism, 335-bp long PCR products were incubated with 10 U *AciI* at 37 °C for 1 h, yielding 254-bp and 81-bp long fragments for the C allele and a noncut 335-bp long fragment for the T allele. The digests were separated by agarose gel electrophoresis (2.5%) and imaged by UV illumination using ethidium bromide staining.

We used genotyping assays based on TaqMan technology (Applied Biosystems, Foster City, CA, USA) to detect rs1532278 C/T (C_1522420_1_) and rs2279590 C/T (C_1842470_20) SNPs. TaqMan genotyping assay (1 ×), TaqMan genotyping PCR master mix (1 ×), and DNA sample (0.8 µg/ml) were used in the assays, and the reactions were carried out on a StepOnePlus Real-Time PCR Instrument (Applied Biosystems, Singapore). The allelic discrimination method was employed on the StepOne v2.3 program (Applied Biosystems).

### Statistical analysis

The continuous variables, as well as the interval variable VFS, were presented as means ± SD, and median and Q1-Q3 quartiles. Normal distribution was checked applying Kolmogorov–Smirnov test. Data that follow a normal distribution were compared using the one-way ANOVA test, while Kruskal–Wallis H test, and the post-hoc Conover’s formula [24] were used to compare data that did not follow a normal distribution. Comparison of two groups was carried out using the Mann–Whitney U test. Categorical variables were shown as percentages and frequencies or ratios, and Pearson’s chi-squared test was used to compare study groups for these variables. Linkage disequilibrium (LD) analysis was carried out using the confidence intervals to define blocks in Haploview [25]. The odds ratio (OR) values and their respective 95% confidence intervals (CIs) were determined to show any possible association of alleles and genotypes for *CLU* with PEG or PEX. These parameters for genotypes were corrected for age and sex using the SNPstats web tool [26]. Pearson's correlation was used to calculate the correlation coefficients for the parameters obtained in this study. The possible risk parameters for PEG or PEX were sought using logistic regression model with the backward selection. Collinearity analysis was carried out before logistic regression and a goodness-of-fit test (Hosmer–Lemeshow) was done to check for the calibration of the logistic regression models. The Genetic Association Study Power Calculator [27] was employed to determine the adequacy of the sample size. The observed minimum power was found to be 93.6% for rs2279590 and rs11136000, 93.5% for rs1532278, and 74% for rs3087554. A power value > 70% was considered acceptable. These statistical analyses were operated using SPSS version 25.0 (SPSS Inc., Chicago, IL, USA) and a *P* value < 0.05 was accepted as the level of statistical significance, unless indicated otherwise. Bonferroni correction at α/n for *P* value was applied for multiple testing.

## Results

The demographic information of the subjects is given in Table 1. The study groups did not differ from each other in terms of age, sex, and the presence of cardiovascular disease, systemic hypertension, diabetes mellitus, or smoking. Visual field score (VFS), IOP, mean deviation (MD), and pattern standard deviation (PSD) parameters were recorded as previously described [28] and are also shown in Table 1.

**Table 1:** Demographic and clinical information of patients with pseudoexfoliative glaucoma (PEG), patients with pseudoexfoliation syndrome (PEX), and the controls

		**PEG (*n* = 239)**	**PEX (*n* = 240)**	**Control (*n* = 240)**	***P***
Age (years),	mean ± SD	71.0 ± 7.9	70.2 ± 6.8	69.7 ± 8.5	.178
	median (Q1-Q3)	72 (66–77)	69 (66–75)	69 (65–76)	
Gender					
male/female, *n*		136/103	122/118	116/124	.155
male %		56.9	50.8	48.3	
Systemic hypertension, *n* (%)		113 (47.3)	113 (47.1)	118 (49.2)	.881
Diabetes, *n* (%)		57 (23.8)	63 (26.3)	61 (25.4)	.828
Cardiovascular disease, *n* (%)		62 (25.9)	70 (29.2)	73 (30.4)	.535
Smokers, *n* (%)		33 (13.8)	32 (13.3)	36 (15.0)	.864
Clinical visual characteristics					
Intraocular pressure (IOP, mmHg),	mean ± SD	21.7 ± 5.3	20.1 ± 5.4	19.7 ± 5.6	< .001
	median (Q1-Q3)	21.0 (18.0 – 24.0)	19.0 (16.0 – 23.0)	19.0 (16.0 – 23.0)	
Visual Field Score (VFS),	mean ± SD	2.61 ± 0.80	1.00 ± 0.00	1.00 ± 0.00	< .001
	median (Q1-Q3)	2.0 (2.0 – 3.0)	1.0 (1.0 – 1.0)	1.0 (1.0 – 1.0)	
Mean deviation (MD),	mean ± SD	-7.08 ± 6.48	-1.23 ± 2.29	-0.98 ± 0.68	< .001
	median (Q1-Q3)	-4.00 (-8.2 – -2.8)	-0.90 (-1.5 – -0.5)	-0.80 (-1.4 – -0.5)	
Pattern standard deviation (PSD),	mean ± SD	4.93 ± 2.98	1.52 ± 0.72	1.45 ± 0.25	< .001
	median (Q1-Q3)	4.10 (2.8 – 6.1)	1.40 (1.3 – 1.6)	1.50 (1.2 – 1.6)	

Continuous variables as well as VFS, an interval variable, were expressed as mean ± SD in the first row, and median (Q1-Q3 quartiles) in the second row, and were compared using the Kruskal–Wallis H test. Categorical variables were expressed as frequencies and percentages, and were compared using the Pearson chi-squared test. *P* value is for comparison of PEG, PEX and control groups

In the present study, all four SNPs were successfully genotyped and Hardy–Weinberg equilibrium was maintained in both patients and controls. The genotype and allele frequencies of patients and controls for four SNPs of *CLU*, rs2279590 C/T, rs11136000 C/T, rs1532278 C/T and rs3087554 T/C, are given in Table 2. The genotype or allele frequencies did not significantly differ from each other in the study population.

**Table 2:** Genotype and allele frequencies of *CLU* rs2279590 C/T, rs11136000 C/T, rs1532278 C/T, and rs3087554 T/C SNPs in patients with PEG, patients with PEX, and the controls

SNP	Genotypes/Alleles	PEG (*n* = 239)	PEX (*n* = 240)	Control (*n* = 240)	Genetic model	Adjusted OR (95% CI)	*P*
**rs2279590 C/T**	**Genotypes, n (%)**				Recessive	0.88^a^ (0.56–1.38) 0.84^b^ (0.54–1.33) 1.10^c^ (0.69–1.76)	.570^a^ .460^b^ .690^c^
	**CC**	97 (40.6)	86 (35.8)	92 (38.3)			
	**CT**	97 (40.6)	110 (45.8)	97 (40.4)	Dominant	0.94^a^ (0.65–1.36) 1.12^b^ (0.77–1.62) 0.84^c^ (0.58–1.21)	.730^a^ .550^b^ .340^c^
	**TT**	45 (18.8)	44 (18.3)	51 (21.2)			
	**Alleles, n (%)**						
	**C**	291 (60.9)	282 (58.8)	281 (58.5)		0.91^a^ (0.70–1.18) 0.99^b^ (0.77–1.28) 0.92^c^ (0.71–1.19)	.460^a^ > .999 .500^c^
	**T**	187 (39.1)	198 (41.2)	199 (41.5)			
**rs11136000 C/T**	**Genotypes, n (%)**				Recessive	0.78^a^ (0.49–1.22) 0.77^b^ (0.49–1.22) 1.06^c^ (0.66–1.71)	.270^a^ .270^b^ .810^c^
	**CC**	99 (41.4)	91 (37.9)	92 (38.3)			
	**CT**	98 (41.0)	107 (44.6)	96 (40.0)	Dominant	0.92^a^ (0.63–1.32) 1.02^b^ (0.71–1.48) 0.88^c^ (0.61–1.27)	.640^a^ .900^b^ .490^c^
	**TT**	42 (17.6)	42 (17.5)	52 (21.7)			
	**Alleles, n (%)**						
	**C**	296 (61.9)	289 (60.2)	280 (58.3)		0.86^a^ (0.66–1.12) 0.93^b^ (0.72–1.20) 0.93^c^ (0.72–1.21)	.260^a^ .550^b^ .590^c^
	**T**	182 (38.1)	191 (39.8)	200 (41.7)			
**rs1532278 C/T**	**Genotypes, n (%)**				Recessive	0.80^a^ (0.51–1.26) 0.79^b^ (0.50–1.25) 1.06^c^ (0.66–1.71)	.340^a^ .320^b^ .800^c^
	**CC**	99 (41.4)	91 (37.9)	93 (38.8)			
	**CT**	98 (41.0)	107 (44.6)	96 (40.0)	Dominant	0.93^a^ (0.64–1.34) 1.04^b^ (0.72–1.50) 0.88^c^ (0.61–1.27)	.680^a^ .840^b^ .490^c^
	**TT**	42 (17.6)	42 (17.5)	51 (21.2)			
	**Alleles, n (%)**						
	**C**	296 (61.9)	289 (60.2)	282 (58.8)		0.88^a^ (0.68–1.14) 0.94^b^ (0.73–1.22) 0.93^c^ (0.72–1.21)	.320^a^ .650^b^ .590^c^
	**T**	182 (38.1)	191 (39.8)	198 (41.2)			
**rs3087554 T/C**	**Genotypes, n (%)**				Recessive	1.65^a^ (0.57–4.76) 2.43^b^ (0.92–6.44) 0.63^c^ (0.27–1.49)	.350^a^ .060^b^ .290^c^
	**TT**	166 (69.5)	170 (70.8)	180 (75.0)			
	**TC**	64 (26.8)	56 (23.3)	54 (22.5)	Dominant	1.33^a^ (0.89–2.00) 1.23^b^ (0.82–1.84) 1.06^c^ (0.71–1.56)	.160^a^ .320^b^ .790^c^
	**CC**	9 (3.8)	14 (5.8)	6 (2.5)			
	**Alleles, n (%)**						
	**T**	396 (82.8)	396 (82.5)	414 (86.2)		1.30^a^ (0.91–1.85) 1.33^b^ (0.94–1.89) 0.98^c^ (0.70–1.36)	.140^a^ .110^b^ > .999^c^
	**C**	82 (17.2)	84 (17.5)	66 (13.8)			

*OR * Odds ratio, *CI * Cnfidence interval, *C * Cytosine, *T * Thymine. ^a^ PEG vs. Control, ^b^ PEX vs. Control, ^c^ PEG vs. PEX. Age- and sex-corrected OR, CI and *P* values for genotypes were calculated using SNPstats web tool. Recessive model; TT vs. CT + CC for rs2279590 C/T, rs11136000 C/T, rs1532278 C/T; CC vs. TC + TT for rs3087554 T/C; Dominant model; TT + CT vs. CC rs2279590 C/T, rs11136000 C/T, rs1532278 C/T;CC + TC vs. TT for rs3087554 T/C. OR, CI and *P* value for the allele frequency was calculated using the χ^2^ test as T vs. C for rs2279590 C/T, rs11136000 C/T, rs1532278 C/T and C vs. T for rs3087554 T/C. Bonferroni corrected significance cut-off value was .01 for genotypes, and .017 for allele frequency

**Fig. 1:**
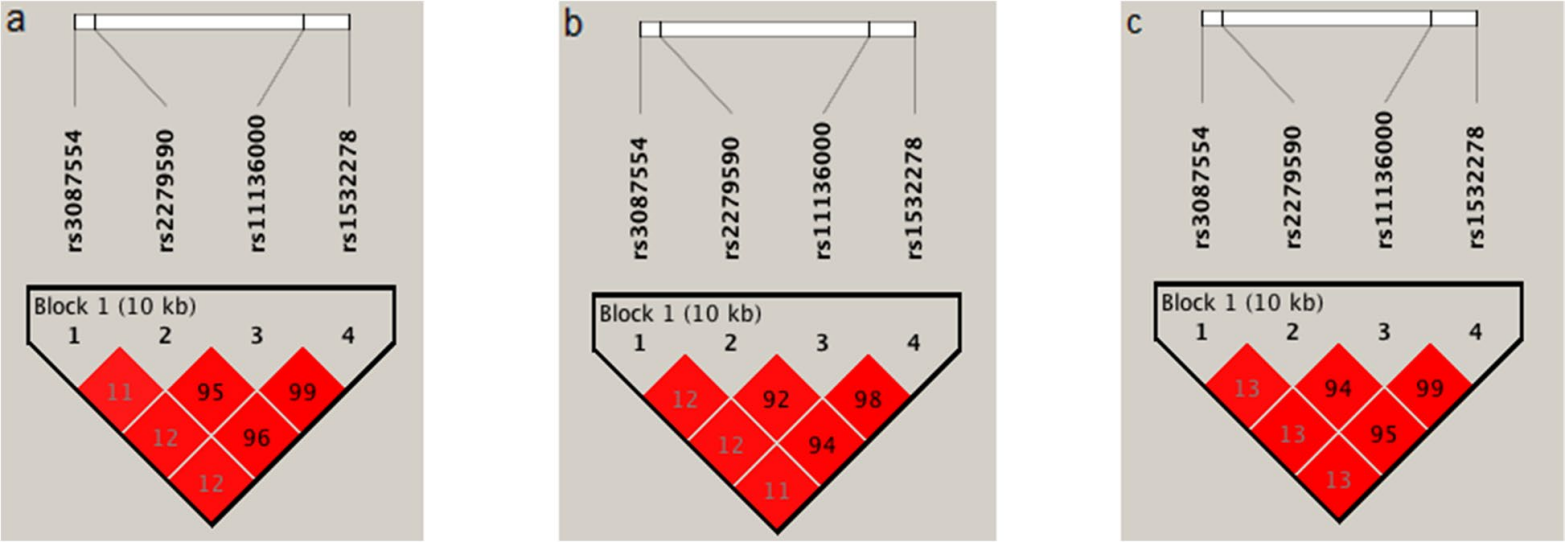
Linkage disequilibrium plot of the 4 *CLU* SNPs analyzed in this study. The numbers given within the diamonds represent the r^2^ values. **a** Pseudoexfoliative glaucoma (PEG) group vs. controls **b**) Pseudoexfoliation syndrome patients without glaucoma (PEX) vs. controls. **c** PEG vs. PEX

LD analysis across *CLU* using the four analyzed SNPs showed that they were included in one haplotype block in both PEG and PEX groups. There was strong LD between the three SNPs, rs2279590, rs11136000, and rs1532278, as shown in Fig. 1. Three haplotypes for PEG and 4 haplotypes for PEX defined by these SNPs were identified. None of the haplotypes was significantly associated with PEG or PEX (*P* > 0.05, Table 3), and their distributions between the two disease groups were similar. The combined genotype analysis also did not give any significant output (data not shown).

**Table 3:** Haplotype associations of *CLU* with PEG and PEX

Haplotype ^a^	PEG—Control			PEX—Control			PEG—PEX		
	Frequency	Chi square	*P*	Frequency	Chi square	*P*	Frequency	Chi square	*P*
1 T-C–C-C	0.441, 0.443	0.004	.947	0.412, 0.442	0.923	.337	0.440, 0.411	0.839	.360
2 T-T-T-T	0.380, 0.406	0.665	.415	0.390, 0.406	0.230	.632	0.380, 0.391	0.120	.729
3 C–C-C–C	0.168, 0.134	2.138	.144	0.172, 0.135	2.523	.112	0.168, 0.172	0.025	.875
4 T-T-C–C	-	-	-	0.015, 0.006	1.613	.204	0.008, 0.015	0.928	.335

^a^ Haplotype order: rs3087554, rs2279590, rs11136000, rs1532278

The analysis of the relationship between visual parameters (VFS, IOP, PSD, and MD) and demographic characteristics and *CLU* SNPs revealed that there was a significant correlation between VFS and IOP (r = 0.156, *P* = .000), MD (r = -0.912, *P* = .000), and PSD (r = 0.891, *P* = .000). In addition, IOP was correlated with MD (r = -0.144, *P* = .000) and PSD (r = 0.140, *P* = .000). Moreover, MD and PSD were also correlated with each other (r = -0.848, *P* = .000).

Stratification analysis of the IOP values of patients with PEG, PEX and control groups with respect to *CLU* genotypes is shown in Table 4. For all SNPs analyzed, patients with PEG with the homozygote wild type genotypes had significantly higher IOP values than controls with the same genotype. On the other hand, IOP values of patients with PEG carrying the homozygote polymorphic genotypes did not differ significantly from that of control subjects with the same genotype. Moreover, patients with PEX had significantly higher IOP values when they had the homozygote polymorphic (TT) genotype for rs2279590, rs11136000 or rs1532278 than patients with PEX with the CT or CC genotypes in a recessive model (*P* = .009, *P* = .007, *P* = .010, respectively; Table 4). These results suggest that homozygote polymorphic genotypes of *CLU* rs2279590, rs11136000 and rs1532278 SNPs might confer higher susceptibility of patients with PEX to develop PEG. Hence, there was no significant difference in the IOP values of patients with PEG and PEX when they had the homozygote polymorphic genotypes. We would like to remind that all PEG cases were on IOP-lowering medications. Stratification analysis of other visual parameters did not reveal any significant difference with respect to genotypes (data not shown).

**Table 4:** Stratification of IOP values of PEG, PEX and control groups with respect to *CLU* rs2279590 C/T, rs11136000 C/T, rs1532278 C/T, and rs3087554 T/C genotypes in a recessive model

	**Genotype**	**PEG (*n* = 239)**	**PEX (*n* = 240)**	**Control (*n* = 240)**	***P***
**rs2279590 C/T**	CC	21.9 ± 5.0 21.0 (18.0 – 24.0)	20.6 ± 5.6 19.5 (18.0 – 23.0)	19.2 ± 4.8 19.0 (16.0 – 22.0)	.002 ^a^, < .001^b^, .153 ^c^, .046 ^d^
	CT	21.2 ± 4.9 21.0 (18.0 – 24.0)	19.0 ± 4.5 18.0 (18.0 – 22.0)	19.6 ± 5.9 18.0 (16.0 – 23.0)	.005 ^a^, .011 ^b^, .658 ^c^, .002 ^d^
	TT	22.4 ± 6.7 21.5 (18.0 – 25.0)	22.3 ± 6.6 21.5 (18.0 – 26.0)	21.0 ± 6.2 19.0 (16.5 – 25.0)	.363^a^
	P*	.603	.009	.181	
**rs11136000 C/T**	CC	21.8 ± 5.0 21.0 (18.0 – 24.0)	20.5 ± 5.6 19.5 (17.0 – 23.0)	19.3 ± 4.8 19.0 (16.0 – 22.0)	.005 ^a^, .001^b^, .189 ^c^, .056 ^d^
	CT	21.4 ± 4.9 21.0 (18.0 – 24.0)	18.9 ± 4.4 18.0 (16.0 – 22.0)	19.6 ± 5.9 18.0 (16.0 – 23.0)	.002 ^a^, .005 ^b^,.629 ^c^, .001 ^d^
	TT	22.3 ± 6.9 20.5 (18.0 – 25.0)	22.4 ± 6.6 22.0 (18.0 – 26.0)	20.9 ± 6.2 19.0 (16.0 – 25.0)	.322 ^a^
	P*	.819	.007	.241	
**rs1532278 C/T**	CC	21.8 ± 5.0 21.0 (18.0 – 24.0)	20.5 ± 5.6 20.0 (17.5 – 23.0)	19.3 ± 4.8 19.0 (16.0 – 22.0)	.005 ^a^, .001 ^b^, .177 ^c^, .059 ^d^
	CT	21.4 ± 4.9 21.0 (18.0 – 24.0)	18.9 ± 4.4 18.0 (16.0 – 22.0)	19.6 ± 5.9 18.0 (16.0 – 23.0)	.002 ^a^, .005 ^b^, .651 ^c^, .001 ^d^
	TT	22.3 ± 6.9 20.5 (18.0 – 25.0)	22.4 ± 6.7 21.5 (18.0 – 26.0)	20.9 ± 6.2 19.0 (16.0 – 25.0)	.361 ^a^
	P*	.819	.010	.248	
**rs3087554 T/C**	TT	21.7 ± 5.8 21.0 (18.0 – 24.0)	20.4 ± 5.6 19.0 (17.0 – 23.0)	19.9 ± 5.8 19.0 (16.0 – 23.5)	.007 ^a^, .002 ^b^, .272 ^c^, .044 ^d^
	TC	21.8 ± 4.4 22.0 (19.0 – 23.0)	18.9 ± 5.0 18.0 (16.0 – 21.0)	19.5 ± 5.1 18.0 (16.0 – 22.0)	< .001 ^a^, .005 ^b^, .428 ^c^, .000 ^d^
	CC	21.6 ± 3.8 20.0 (19.0 – 26.0)	21.0 ± 5.0 20.0 (20.0 – 22.0)	18.2 ± 3.0 18.0 (16.0 – 21.0)	.305 ^e^
	P*	.934	.306	.557	

Data were expressed as mean ± SD in the first row, and median (Q1-Q3 quartiles) in the second row. ^a^ Comparison of three groups (PEG vs. PEX vs. Control) by Kruskal–Wallis test. ^b^ PEG vs. Control; ^c^ PEX vs. Control; ^d^ PEG vs. PEX by Conover’s formula. ^e^ Comparison of three groups (PEG vs. PEX vs. Control) by One-Way ANOVA. * *P* value is for recessive model; TT vs. CT + CC for rs2279590 C/T, rs11136000 C/T, rs1532278 C/T; CC vs. TC + TT for rs3087554 T/C by Mann–Whitney *U* test. Bonferroni corrected significance cut-off value was .0125

The influence of age, sex, cardiovascular disease, systemic hypertension, diabetes, smoking, clinical visual parameters, and *CLU* SNPs on PEX or PEG risk were assessed with the help of logistic regression analysis. There was collinearity between the *CLU* SNPs rs11136000, rs1532278, and rs2279590, and the visual parameters IOP, VFS, MD, and PSD. Age (being ≥ 70 years old) was significantly associated with PEG (OR = 4.000, 95% CI = 1.412 – 11.364, *P* = .009) when MD was in the model for PEG versus controls analysis. In addition, MD was also associated with PEG (OR = 0.002, 95% CI = 0.000 – 0.013, *P* = .000). Furthermore, regression analysis for PEG versus PEX cases revealed that IOP (OR = 1.062, 95% CI = 1.021 – 1.104, *P* = .002), and being 70 years old or over (OR = 2.062, 95% CI = 1.395 – 3.058, *P* = .000) were significant predictors for developing PEG in PEX cases. The calibration of these models was satisfactory. In contrast, none of the tested parameters was associated significantly with the development of PEX and none of the *CLU* SNPs were significantly associated with PEX or PEG.

## Discussion

Previous studies have found genetic variations on lysyl oxidase-like 1 (*LOXL*1) gene to be associated with PEX or PEG [29]; however, it was concluded that these variants were not useful to discriminate those PEX patients who are more susceptible to develop PEG [30]. CLU has formerly been implicated to take a part in the pathophysiological mechanisms of PEG and PEX [15, 31]. Interestingly, the expression of *CLU* in PEX patients is low despite the oxidative stress, which should actually upregulate the expression. This observation suggests that some other factors may cause the lower CLU levels in PEX patients. Therefore, we evaluated whether *CLU* genetic polymorphisms that cause a low level of protein expression had any effect on the development of PEX and/or PEG or on IOP levels.

*CLU* is the most richly expressed gene in the normal iris tissue [32]. Iris and cornea make up the anterior chamber angle, which comprises of the trabecular meshwork that is important for the drainage of the aqueous humor. *CLU* genetic variations that cause decreased expression level can potentially influence the IOP via leading to a locally diminished chaperone activity and decreased turnover of extracellular matrix. We observed increased IOP values in PEX cases carrying the polymorphic TT genotype for the three SNPs analyzed in this study, rs2279590, rs11136000 and rs1532278 compared with patients carrying the CT or CC genotypes. This finding can potentially be utilized as a biomarker that can help identify those patients with PEX who are more susceptible to develop PEG. We are not aware of any previous studies showing a relationship between *CLU* SNPs and IOP.

Elevation of IOP in patients with PEX may represent an earlier stage of PEG development. In the advanced stages, retinal ganglion cells and optic nerve degenerate via apoptotic mechanisms due to the increased IOP levels. In our previous study, we have observed elevated CLU concentration in aqueous humor of patients with PEG than in patients with PEX [33]. This makes sense because CLU is a molecular chaperone that provides either the continuity of life or the programmed death of the cell depending on the cellular stress level and could have been induced in the pathological millieu of increased oxidative stress and degenerating retinal ganglion cells.

We also analyzed the direct association between *CLU* minor alleles and PEX or PEG status. *CLU* rs2279590 C/T SNP has previously been reported to have a role in *CLU* expression level [15], and was significantly associated with PEX [15, 19]. These studies were carried out on Indian [15] and German populations [19], respectively, thus genetic background was different from our study population. Moreover, the sample size was small in the study of Padhy et al. [15]. On the other hand, we did not observe this variant to be correlated with the risk of developing PEG or PEX in the Turkish population. Likewise, some other studies have also detected no association of this SNP with PEX [20, 21] or PEG [21]. It is well known that the distribution pattern of alleles generally markedly differs among populations. Therefore, we compared the minor allele frequencies in the control group of this study with those in different populations that have previously been analyzed [15, 19–21, 23, 34–41] (Table 5). The T allele frequency was reported to be 0.294 in TOPMED, 0.395 in ALFA Project, and 0.211 in the PEGE_STUDY [16]. The rs2279590T allele frequency detected in the present study (0.415) is similar to that determined by Alaylıoğlu et al. [40] for the Turkish population (0.367), and it is also close to those found in French [41] (0.410) and Russian [34] (0.392) populations.

**Table 5:** *CLU* rs2279590 C/T, rs11136000 C/T, rs1532278 C/T, and rs3087554 T/C minor allele frequencies reported in healthy control groups of different populations

Reference	Population	*CLU* minor allele frequency in the healthy control population			
		**rs2279590 T**	**rs11136000 T**	**rs1532278 T**	**rs3087554 C**
**This study**	**Turkish**	**0.415**	**0.417**	**0.413**	**0.138**
Kılıçarslan et al*.*, 2017 [22]	Turkish	-	0.405	-	-
Alaylıoǧlu et al*.*, 2016 [40]	Turkish	0.367	0.367	-	-
Lambert et al*.*, 2009 [41]	French	0.410	0.380	-	
Bocharova et al*.*, 2018 [34]	Russian	0.392	-	0.380	-
Krumbiegel et al*.*, 2009 [19]	German	0.347	0.660	-	0.204
Fan et al*.*, 2015 [20]	American	0.320	0.360	-	0.180
	Israeli	0.350	0.380	-	0.150
Dubey et al*.*, 2015 [21]	South Indian	0.263	0.279	-	0.340
Ma et al*.,* 2019 [23]	Uygur	0.302	-	-	-
Padhy et al*.*, 2014 [15]	Indian	0.480	-	-	0.540
Mullan et al*.*, 2013 [14]	North Irish	-	0.412	-	-
Burdon et al*.*, 2008 [18]	Australian (Blue Mountain)	-	0.400	-	0.180
Harold et al*.*, 2009 [42]	European and American	-	0.400	-	-
Yu et al*.*, 2010 [35]	Chinese Han	0.183	0.193	-	-
Yu et al*.*, 2013 [36]	Chinese Han	0.202	-	0.211	0.237
Lu et al*.*, 2014 [37]	Chinese Han	0.174	0.186	0.190	-
Ma et al*.*, 2011 [43]	Chinese Han	-	0.238	-	-
Cai et al*.*, 2016 [44]	Chinese Han	-	0.219	-	-
Xian et al*.*, 2017 [45]	Chinese Han	-	0.183	-	-
Li et al*.*, 2015 [46]	Chinese Han	-	-	0.563	-
Chen et al*.*, 2012 [38]	South Chinese	0.234	0.240	-	-
Komatsu et al*.*, 2011 [39]	Japanese	0.234	-	-	-
Lin et al*.*, 2012 [47]	Taiwanese	-	0.226	-	0.257
Aghajanpour-Mir et al*.*, 2019 [48]	Iranian (age ≥ 75 years)	-	0.351	-	-
	Iranian (age < 75 years)	-	0.336	-	-
Kuot et al*.*, 2012 [49]	Australian	-	-	0.400	0.170

*C* Cytosine, T Thymine

*CLU* rs11136000 C/T SNP plays a role in *CLU* expression levels. The minor T allele has been associated with lower plasma CLU levels in control individuals in previous studies on Alzheimer's disease [13, 14]. We did not observe any significant effect of this SNP on PEX or PEG status in this study, consistent with other reports, which have not detected a significant association between rs11136000T and PEX or PEG [18–22]. The frequency of the minor rs11136000T allele in the control group of the present study (0.417) is similar to that found in the studies of Kılıçarslan et al. [22] (0.405) and Alaylıoğlu et al. [40] (0.367), which were also carried out on Turkish population (Table 5). These values are also similar to those observed in the North Irish [14], Australian [18], European [42], Israeli [20], and French [41] populations. On the other hand, the German population has a higher frequency [19], and the American [20], South Indian [21], Chinese Han [35, 37, 43–45], South Chinese [38], Taiwanese [47], and Iranian [48] populations have lower frequencies (Table 5). The frequency of the T allele was 0.429 in TOPMED, 0.398 in ALFA Project, and 0.427 in PAGE_STUDY [16].

This is the first time *CLU* rs1532278 C/T SNP has been studied in PEX, but no correlation with PEG or PEX was detected. There are similarities in the pathophysiological courses of PEX and Alzheimer’s disease and *CLU* rs1532278 variant has been reported to be significantly associated with late onset Alzheimer’s disease in a large cohort study [50]. As shown in Table 5, the minor allele frequency detected in the control group of this study was close to that estimated for the Australian [49] and Russian populations [34], but was different from that found in Chinese Han population [36, 37, 46]. The T allele frequency was reported as 0.311in TOPMED, 0.380 in ALFA Project, and 0.250 in PAGE_STUDY [16].

In the present study, the 3’UTR variant *CLU* rs3087554 T/C SNP was not found to be correlated with PEG or PEX, consistent with the results of other reports regarding PEX [15, 18–21] or PEG [15, 21]. The present study is the first time rs3087554 T/C allele frequency has been evaluated in the Turkish population, and the estimated minor allele frequency (0.138) was found to be similar to that obtained for the Israeli [20], American [20] and Australian populations [18], but much lower than those in other populations [15, 19, 21, 36, 47, 49] (Table 5). The frequency of the C allele was 0.201 in TOPMED and 0.180 in ALFA Project [16].

Some limitations associated with this manuscript could be addressed. We cannot rule out the possibility that other genetic variations of *CLU* gene, not examined in the present study, could be directly related to disease risk. Thus, we suggest that new studies focusing on different *CLU* genetic variants on different ethnic backgrounds are still required.

## Conclusion

Overall, this study evaluated the possible role played by *CLU* genetic variants in the development of PEG or PEX. Even though the analyzed *CLU* SNPs were not directly associated with PEG or PEX in the Turkish population, we observed evidence that three SNPs in *CLU* gene might induce IOP elevation in individuals with PEX, conferring a greater susceptibility to develop PEG. Further studies focusing on molecular mechanisms leading to IOP rise in PEX cases have the potential to expand these findings.

## Data Availability

The datasets used and/or analyzed during the current study are available from the corresponding author on reasonable request.
